# Supplementary material for: Computational investigation unveils pathogenic LIG3 non-synonymous mutations and therapeutic targets in acute myeloid leukemia
Source: PLoS One. 2025 Jun 10;20(6):e0320550. doi: 10.1371/journal.pone.0320550 (PMC12151348; doi:10.1371/journal.pone.0320550)
Supplement: S1 Table — (DOCX) [file pone.0320550.s001.docx]

**S1 Table:** List of 132 nsSNPs + Somatic variants of the *LIG3* gene in the NCBI dbSNP database.

| Serial No. | rs ID | Amino acid change and position |
| --- | --- | --- |
|  | rs3744356 | R224G |
|  | rs139474938 | A901T |
|  | rs140462567 | R1003Q |
|  | rs200974999 | R942Q |
|  | rs370882864 | R343Q |
|  | rs537076655 | R151Q |
|  | rs1076992 | R30C |
|  | rs3136025 | R867H |
|  | rs36021499 | R100C |
|  | rs141095047 | V116I |
|  | rs143109771 | S424L |
|  | rs144452860 | R954H |
|  | rs145709942 | R806H |
|  | rs145715449 | E158K |
|  | rs145992710 | R528H |
|  | rs147386726 | D543N |
|  | rs148073351 | D323N |
|  | rs148247013 | R528S |
|  | rs149175581 | R264Q |
|  | rs151123339 | R92W |
|  | rs151264739 | R1001Q |
|  | rs187881525 | R25Q |
|  | rs188396172 | R867S |
|  | rs199806002 | R291Q |
|  | rs200267761 | R608K |
|  | rs201706144 | R383W |
|  | rs202243099 | R224Q |
|  | rs368652393 | P366T |
|  | rs372375745 | R635Q |
|  | rs376449090 | G301S |
|  | rs532596955 | M503I |
|  | rs547119482 | A458T |
|  | rs549899201 | D925A |
|  | rs559259641 | P465L |
|  | rs559299489 | G940R |
|  | rs559318643 | R740H |
|  | rs564927633 | I326M |
|  | rs567712080 | R445H |
|  | rs572202786 | R921W |
|  | rs577965471 | S130F |
|  | rs746442180 | M496T |
|  | rs746484841 | G851A |
|  | rs747002495 | H428Y |
|  | rs747853708 | R118Q |
|  | rs748570556 | R671Q |
|  | rs748659769 | R67H |
|  | rs749058293 | Q586R |
|  | rs749670812 | R14H |
|  | rs750136278 | G313R |
|  | rs750258790 | A998E |
|  | rs750517714 | A643S |
|  | rs751006081 | E881K |
|  | rs751540797 | R25G |
|  | rs752902294 | G206D |
|  | rs755498333 | V978M |
|  | rs756649783 | R957S |
|  | rs757797167 | D717N |
|  | rs757932526 | S491F |
|  | rs758686695 | G706S |
|  | rs758823264 | R100H |
|  | rs758886375 | P945T |
|  | rs759246540 | S444L |
|  | rs760754334 | R648G |
|  | rs761685440 | S476L |
|  | rs761808558 | P55R |
|  | rs762809759 | D302N |
|  | rs764607272 | R627Q |
|  | rs764868558 | V748L |
|  | rs765117391 | R291W |
|  | rs765524171 | V473I |
|  | rs765806516 | Y699C |
|  | rs768909567 | N280S |
|  | rs769573144 | R332G |
|  | rs770559358 | P126H |
|  | rs770579198 | R614G |
|  | rs770871827 | Y944D |
|  | rs771323148 | R267Q |
|  | rs771903503 | P366R |
|  | rs773669956 | V781M |
|  | rs773813748 | D679Y |
|  | rs773949692 | D798A |
|  | rs776731503 | E173K |
|  | rs776944632 | P48L |
|  | rs779188644 | R671G |
|  | rs779881244 | A215T |
|  | rs780735155 | V533I |
|  | rs780940582 | S847G |
|  | rs781345619 | A271T |
|  | rs947282793 | T796K |
|  | rs967586901 | I710V |
|  | rs1007072275 | A987E |
|  | rs1042320819 | D965H |
|  | rs1162766165 | M972I |
|  | rs1167454497 | T281A |
|  | rs1173645231 | T12N |
|  | rs1194206854 | S294L |
|  | rs1202657275 | E390D |
|  | rs1216090765 | D596Y |
|  | rs1225851538 | S792L |
|  | rs1226387269 | S861L |
|  | rs1229048697 | A488D |
|  | rs1265279288 | G132V |
|  | rs1272966444 | G918R |
|  | rs1289033781 | E783D |
|  | rs1295932202 | D963N |
|  | rs1301120665 | A486V |
|  | rs1305748395 | G165V |
|  | rs1306395065 | A426V |
|  | rs1314034151 | R8H |
|  | rs1318377484 | G859D |
|  | rs1363874124 | P860R |
|  | rs1369910978 | G799R |
|  | rs1370270201 | R957H |
|  | rs1402186516 | G76E |
|  | rs1411966445 | T849P |
|  | rs1430297615 | F585L |
|  | rs1435111560 | P914S |
|  | rs1441563785 | E148D |
|  | rs1444808295 | A458V |
|  | rs1458329738 | R803Q |
|  | rs1459305342 | A68T |
|  | rs1465441935 | M645V |
|  | rs1466278171 | T204A |
|  | rs1597795020 | L381R |
|  | rs1597795703 | A432T |
|  | rs2090622001 | R67C |
|  | rs2090651973 | A215V |
|  | rs2090734484 | Y438C |
|  | rs2090757033 | D521N |
|  | rs2090781376 | S604T |
|  | rs2090840436 | P823S |
|  | rs2090848085 | S863C |
