# Supplementary material for: Computational investigation unveils pathogenic LIG3 non-synonymous mutations and therapeutic targets in acute myeloid leukemia
Source: PLoS One. 2025 Jun 10;20(6):e0320550. doi: 10.1371/journal.pone.0320550 (PMC12151348; doi:10.1371/journal.pone.0320550)
Supplement: S2 Table — (DOCX) [file pone.0320550.s002.docx]

**S2 Table:**  High risk nsSNPs identified by eight computational tools.

| Rs ID | Amino acid change | Predict SNP | PANTHER | SIFT | PHD-SNP | MAPP | SNAP | PolyPhen1 | PolyPhen2 |
| --- | --- | --- | --- | --- | --- | --- | --- | --- | --- |
| rs148247013 | R528C | DL | N | DL | D | DL | E | DL | PD |
| rs765806516 | Y699C | DL | DL | DL | D | N | E | DL | PD |
| rs770579198 | R614G | DL | N | DL | D | DL | E | DL | N |
| rs773669956 | V781M | DL | DL | DL | D | DL | E | DL | PD |
| rs773813748 | D679Y | DL | N | DL | D | DL | E | DL | PD |
| rs779188644 | R671G | DL | N | DL | D | DL | E | DL | N |
| rs1216090765 | D596Y | DL | N | DL | D | DL | E | DL | PD |
| rs1225851538 | S792L | DL | DL | DL | D | DL | E | DL | N |
| rs1229048697 | A488D | DL | DL | DL | D | N | E | DL | PD |
| rs1305748395 | G165V | DL | N | DL | D | N | E | DL | PD |
| rs1369910978 | G799R | DL | N | DL | D | DL | E | DL | PD |
| rs1597795020 | L381R | DL | N | DL | D | DL | E | DL | PD |
| rs1597795703 | A432T | DL | N | DL | D | DL | N | DL | PD |
| rs145709942 | R806H | DL | N | DL | D | DL | E | DL | N |
| rs145992710 | R528H | DL | N | DL | D | DL | E | DL | N |
| rs559299489 | G940R | DL | N | DL | D | N | E | DL | PD |
| rs572202786 | R921W | DL | DL | DL | N | N | E | DL | PD |
| rs1301120665 | A486V | DL | N | DL | D | DL | N | DL | PD |

*DL: deleterious; D: disease; E: effect; PD: probably damaging; N: Neutral*
