# Supplementary material for: Computational investigation unveils pathogenic LIG3 non-synonymous mutations and therapeutic targets in acute myeloid leukemia
Source: PLoS One. 2025 Jun 10;20(6):e0320550. doi: 10.1371/journal.pone.0320550 (PMC12151348; doi:10.1371/journal.pone.0320550)
Supplement: S3 Table — (DOCX) [file pone.0320550.s003.docx]

**S3 Table:** List of nsSNPs affecting protein stability detected using I-Mutant 2.0 and MUpro.

| Rs ID | Mutation | I-Mutant 2.0 | | MUpro | | mCSM |  |
| --- | --- | --- | --- | --- | --- | --- | --- |
|  |  | Stability | ΔΔG (kcal/mol) | Stability | ΔΔG (kcal/mol) | Stability | ΔΔG (kcal/mol) |
| rs148247013 | R528C | D | -2.03 | D | -0.818 | D | -0.284 |
| rs770579198 | R614G | D | -1.32 | D | -1.995 | D | -2.199 |
| rs773669956 | V781M | D | -1.16 | D | -0.933 | D | -1.411 |
| rs779188644 | R671G | D | -1.21 | D | -1.131 | D | -0.524 |
| rs1305748395 | G165V | D | -1.84 | D | -0.476 | D | -0,39 |
| rs1369910978 | G799R | D | -1.89 | D | -0.308 | D | -0.954 |
| rs1597795020 | L381R | D | -1.41 | D | -1.61 | D | -2.012 |
| rs1597795703 | A432T | D | -1.66 | D | -0.652 | D | -1.627 |
| rs145709942 | R806H | D | -1.47 | D | -0.71 | D | -1.355 |
| rs145992710 | R528H | D | -2.14 | D | -1.148 | D | -0.704 |
| rs559299489 | G940R | D | -1.89 | D | -0.341 | D | -0.494 |
| rs572202786 | R921W | D | -0.19 | D | -0.829 | D | -0.332 |

‘D: Decrease’
