# Supplementary material for: Computational investigation unveils pathogenic LIG3 non-synonymous mutations and therapeutic targets in acute myeloid leukemia
Source: PLoS One. 2025 Jun 10;20(6):e0320550. doi: 10.1371/journal.pone.0320550 (PMC12151348; doi:10.1371/journal.pone.0320550)
Supplement: S4 Table — (DOCX) [file pone.0320550.s004.docx]

**S4 Table:** Estimation of LIG3 phosphorylation sites utilizing NetPhos 3.1 in both wild-type and mutant-type variants.

| **Netphos 3.1** | **Wild Type** | | | | | **Netphos 3.1** | **Mutant Residues** | | |
| --- | --- | --- | --- | --- | --- | --- | --- | --- | --- |
| **Serine (S)** | **Position** | **Score** | | | **Kinase** | **Serine (S)** | **Position** | **Score** | **Kinase** |
|  | 2 S | 0.551 | | | DNAPK |  | 2 S | 0.551 | DNAPK |
|  | 17 S | 0.998 | | | unsp |  | 17 S | 0.998 | unsp |
|  | 17 S | 0.737 | | | PKA |  | 17 S | 0.737 | PKA |
|  | 17 S | 0.535 | | | PKG |  | 17 S | 0.535 | PKG |
|  | 17 S | 0.510 | | | RSK |  | 17 S | 0.510 | RSK |
|  | 36 S | 0.584 | | | DNAPK |  | 36 S | 0.584 | DNAPK |
|  | 36 S | 0.573 | | | PKA |  | 36 S | 0.573 | PKA |
|  | 39 S | 0.877 | | | unsp |  | 39 S | 0.877 | unsp |
|  | 62 S | 0.510 | | | cdc2 |  | 62 S | 0.510 | cdc2 |
|  | 66 S | 0.603 | | | PKA |  | 66 S | 0.603 | PKA |
|  | 128 S | 0.543 | | | CKI |  | 128 S | 0.543 | CKI |
|  | 130 S | 0.520 | | | CKII |  | 130 S | 0.520 | CKII |
|  | 130 S | 0.501 | | | CKI |  | 130 S | 0.501 | CKI |
|  | 185 S | 0.894 | | | PKC |  | 185 S | 0.894 | PKC |
|  | 186 S | 0.557 | | | PKC |  | 186 S | 0.557 | PKC |
|  | 210 S | 0.553 | | | p38MAPK |  | 210 S | 0.553 | p38MAPK |
|  | 210 S | 0.543 | | | cdk5 |  | 210 S | 0.543 | cdk5 |
|  | 220 S | 0.692 | | | PKC |  | 220 S | 0.692 | PKC |
|  | 227 S | 0.678 | | | PKC |  | 227 S | 0.678 | PKC |
|  | 227 S | 0.505 | | | PKG |  | 227 S | 0.505 | PKG |
|  | 230 S | 0.734 | | | PKC |  | 230 S | 0.734 | PKC |
|  | 241 S | 0.545 | | | cdc2 |  | 241 S | 0.545 | cdc2 |
|  | 242 S | 0.580 | | | cdk5 |  | 242 S | 0.580 | cdk5 |
|  | 242 S | 0.567 | | | p38MAPK |  | 242 S | 0.567 | p38MAPK |
|  | 242 S | 0.505 | | | GSK3 |  | 242 S | 0.505 | GSK3 |
|  | 248 S | 0.611 | | | PKG |  | 248 S | 0.611 | PKG |
|  | 250 S | 0.595 | | | RSK |  | 250 S | 0.595 | RSK |
|  | 250 S | 0.582 | | | PKA |  | 250 S | 0.582 | PKA |
|  | 250 S | 0.547 | | | PKG |  | 250 S | 0.547 | PKG |
|  | 251 S | 0.915 | | | PKC |  | 251 S | 0.915 | PKC |
|  | 252 S | 0.997 | | | unsp |  | 252 S | 0.997 | unsp |
|  | 294 S | 0.997 | | | unsp |  | 294 S | 0.997 | unsp |
|  | 294 S | 0.767 | | | PKA |  | 294 S | 0.767 | PKA |
|  | 294 S | 0.678 | | | PKG |  | 294 S | 0.678 | PKG |
|  | 331 S | 0.567 | | | PKC |  | 331 S | 0.567 | PKC |
|  | 361 S | 0.522 | | | cdc2 |  | 361 S | 0.522 | cdc2 |
|  | 363 S | 0.605 | | | cdc2 |  | 363 S | 0.605 | cdc2 |
|  | 385 S | 0.512 | | | PKA |  | 385 S | 0.512 | PKA |
|  | 444 S | | 0.703 | | PKA |  | 444 S | 0.703 | PKA |
|  | 472 S | | 0.697 | | PKA |  | 472 S | 0.697 | PKA |
|  | 472 S | | 0.665 | | PKC |  | 472 S | 0.665 | PKC |
|  | 472 S | | 0.542 | | RSK |  | 472 S | 0.542 | RSK |
|  | 476 S | | 0.510 | | cdc2 |  | 476 S | 0.510 | cdc2 |
|  | 491 S | | 0.590 | | unsp |  | 491 S | 0.787 | unsp |
|  | 505 S | | 0.721 | | unsp |  | 505 S | 0.721 | unsp |
|  | 524 S | | 0.689 | | PKC |  | 524 S | 0.565 | PKC |
|  | 529 S | | 0.791 | | unsp |  | 529 S | 0.678 | PKC |
|  | 529 S | | 0.630 | | PKC |  | 554 S | 0.504 | CKII |
|  | 554 S | | 0.504 | | CKII |  | 604 S | 0.510 | unsp |
|  | 604 S | | 0.510 | | unsp |  | 631 S | 0.606 | PKC |
|  | 631 S | | 0.606 | | PKC |  | 703 S | 0.520 | CKI |
|  | 703 S | | 0.515 | | CKI |  | 709 S | 0.519 | PKA |
|  | 709 S | | 0.519 | | PKA |  | 720 S | 0.809 | PKC |
|  | 720 S | | 0.809 | | PKC |  | 720 S | 0.630 | unsp |
|  | 720 S | | 0.630 | | unsp |  | 720 S | 0.609 | DNAPK |
|  | 720 S | | 0.609 | | DNAPK |  | 751 S | 0.785 | unsp |
|  | 751 S | | 0.785 | | unsp |  | 751 S | 0.565 | PKC |
|  | 751 S | | 0.565 | | PKC |  | 755 S | 0.924 | unsp |
|  | 755 S | | 0.924 | | unsp |  | 790 S | 0.981 | unsp |
|  | 790 S | | 0.981 | | unsp |  | 790 S | 0.597 | CKI |
|  | 790 S | | 0.597 | | CKI |  | 790 S | 0.528 | CKII |
|  | 790 S | | 0.528 | | CKII |  | 792 S | 0.682 | unsp |
|  | 792 S | | 0.682 | | unsp |  | 801 S | 0.955 | unsp |
|  | 801 S | | 0.955 | | unsp |  | 818 S | 0.770 | unsp |
|  | 818 S | | 0.770 | | unsp |  | 832 S | 0.758 | unsp |
|  | 832 S | | 0.758 | | unsp |  | 847 S | 0.593 | CKI |
|  | 847 S | | 0.593 | | CKI |  | 848 S | 0.944 | unsp |
|  | 848 S | | 0.944 | | unsp |  | 853 S | 0.996 | unsp |
|  | 853 S | | 0.996 | | unsp |  | 853 S | 0.502 | CKII |
|  | 853 S | | 0.502 | | CKII |  | 854 S | 0.992 | unsp |
|  | 854 S | | 0.992 | | unsp |  | 861 S | 0.687 | unsp |
|  | 861 S | | 0.687 | | unsp |  | 861 S | 0.523 | cdc2 |
|  | 861 S | | 0.523 | | cdc2 |  | 866 S | 0.992 | unsp |
|  | 866 S | | 0.992 | | unsp |  | 866 S | 0.855 | PKC |
|  | 866 S | | 0.855 | | PKC |  | 871 S | 0.951 | unsp |
|  | 871 S | | 0.951 | | unsp |  | 871 S | 0.733 | PKC |
|  | 871 S | | 0.733 | | PKC |  | 874 S | 0.504 | PKC |
|  | 874 S | | 0.504 | | PKC |  | 874 S | 0.502 | cdc2 |
|  | 874 S | | 0.502 | | cdc2 |  | 876 S | 0.996 | unsp |
|  | 876 S | | 0.996 | | unsp |  | 876 S | 0.828 | PKC |
|  | 876 S | | 0.828 | | PKC |  | 885 S | 0.936 | unsp |
|  | 885 S | | 0.936 | | unsp |  | 887 S | 0.540 | cdc2 |
|  | 887 S | | 0.540 | | cdc2 |  | 900 S | 0.622 | PKG |
|  | 900 S | | 0.622 | | PKG |  | 912 S | 0.631 | PKC |
|  | 912 S | | 0.631 | | PKC |  | 948 S | 0.931 | unsp |
|  | 948 S | | 0.931 | | unsp |  | 948 S | 0.592 | cdc2 |
|  | 948 S | | 0.592 | | cdc2 |  | 981 S | 0.907 | PKC |
|  | 981 S | | 0.907 | | PKC |  | 981 S | 0.686 | unsp |
|  | 981 S | | 0.686 | | unsp |  | 992 S | 0.898 | unsp |
|  | 992 S | | 0.898 | | unsp |  |  |  |  |
|  | 992 S | | 0.898 | | unsp | **Threonine (T)** | 12 T | 0.924 | unsp |
| **Threonine (T)** | 12 T | | 0.924 | | unsp |  | 12 T | 0.924 | unsp |
|  |  | |  | |  |  | 12 T | 0.759 | PKC |
|  | 12 T | | 0.759 | | PKC |  | 41 T | 0.532 | CKII |
|  | 41 T | | 0.532 | | CKII |  | 69 T | 0.784 | PKC |
|  | 69 T | | 0.784 | | PKC |  | 69 T | 0.657 | unsp |
|  | 69 T | | 0.657 | | unsp |  | 102 T | 0.753 | PKC |
|  | 102 T | | 0.753 | | PKC |  | 153 T | 0.970 | unsp |
|  | 153 T | | 0.970 | | unsp |  | 153 T | 0.924 | PKC |
|  | 153 T | | 0.924 | | PKC |  | 154 T | 0.981 | unsp |
|  | 154 T | | 0.981 | | unsp |  | 154 T | 0.726 | PKB |
|  | 154 T | | 0.726 | | PKB |  | 154 T | 0.535 | PKG |
|  | 154 T | | 0.535 | | PKG |  | 154 T | 0.526 | PKC |
|  | 154 T | | 0.526 | | PKC |  | 161 T | 0.692 | CKII |
|  | 161 T | | 0.692 | | CKII |  | 191 T | 0.987 | unsp |
|  | 191 T | | 0.987 | | unsp |  | 191 T | 0.800 | PKC |
|  | 191 T | | 0.800 | | PKC |  | 191 T | 0.532 | p38MAPK |
|  | 191 T | | 0.532 | | p38MAPK |  | 203 T | 0.509 | PKC |
|  | 203 T | | 0.509 | | PKC |  | 204 T | 0.570 | PKC |
|  | 204 T | | 0.570 | | PKC |  | 205 T | 0.517 | PKC |
|  | 205 T | | 0.517 | | PKC |  | 209 T | 0.563 | PKC |
|  | 209 T | | 0.563 | | PKC |  | 221 T | 0.805 | PKC |
|  | 221 T | | 0.805 | | PKC |  | 244 T | 0.942 | unsp |
|  | 244 T | | 0.942 | | unsp |  | 244 T | 0.602 | cdk5 |
|  | 244 T | | 0.602 | | cdk5 |  | 244 T | 0.587 | PKC |
|  | 244 T | | 0.587 | | PKC |  | 244 T | 0.534 | p38MAPK |
|  | 244 T | | 0.534 | | p38MAPK |  | 281 T | 0.517 | PKC |
|  | 281 T | | 0.517 | | PKC |  | 306 T | 0.676 | unsp |
|  | 306 T | | 0.676 | | unsp |  | 306 T | 0.657 | PKC |
|  | 306 T | | 0.657 | | PKC |  | 353 T | 0.927 | unsp |
|  | 353 T | | 0.927 | | unsp |  | 353 T | 0.558 | PKC |
|  | 353 T | | 0.558 | | PKC |  | 373 T | 0.512 | CKII |
|  | 373 T | | 0.512 | | CKII |  | 479 T | 0.624 | p38MAPK |
|  | 479 T | | 0.624 | | p38MAPK |  | 479 T | 0.595 | cdk5 |
|  | 479 T | | 0.595 | | cdk5 |  | 479 T | 0.562 | unsp |
|  | 479 T | | 0.562 | | unsp |  | 568 T | 0.698 | PKC |
|  | 568 T | | 0.698 | | PKC |  | 637 T | 0.596 | PKA |
|  | 637 T | | 0.596 | | PKA |  | 665 T | 0.518 | PKC |
|  | 665 T | | 0.518 | | PKC |  | 689 T | 0.571 | unsp |
|  | 689 T | | 0.571 | | unsp |  | 689 T | 0.504 | CKII |
|  | 689 T | | 0.504 | | CKII |  | 725 T | 0.634 | PKC |
|  | 725 T | | 0.634 | | PKC |  | 727 T | 0.597 | PKC |
|  | 727 T | | 0.597 | | PKC |  | 737 T | 0.637 | PKC |
|  | 737 T | | 0.637 | | PKC |  | 785 T | 0.550 | unsp |
|  | 785 T | | 0.694 | | unsp |  | 785 T | 0.507 | CKII |
|  | 785 T | | 0.515 | | CKII |  | 850 T | 0.614 | CKI |
|  | 850 T | | 0.614 | | CKI |  | 877 T | 0.944 | unsp |
|  | 877 T | | 0.944 | | unsp |  | 877 T | 0.926 | PKC |
|  | 877 T | | 0.926 | | PKC |  | 896 T | 0.821 | unsp |
|  | 896 T | | 0.821 | | unsp |  | 910 T | 0.549 | PKC |
|  | 910 T | | 0.549 | | PKC |  | 927 T | 0.729 | unsp |
|  | 949 T | | 0.597 | | cdk5 |  | 949 T | 0.597 | cdk5 |
|  | 949 T | | 0.515 | | GSK3 |  | 949 T | 0.515 | GSK3 |
|  | 949 T | | | 0.508 | p38MAPK |  | 949 T | 0.508 | p38MAPK |
| **Tyrosine (Y)** | 97 Y | | | 0.871 | unsp | **Tyrosine (Y)** | 97 Y | 0.871 | unsp |
|  | 138 Y | | | 0.666 | unsp |  | 138 Y | 0.666 | unsp |
|  | 279 Y | | | 0.916 | unsp |  | 279 Y | 0.916 | unsp |
|  | 279 Y | | | 0.521 | EGFR |  | 279 Y | 0.521 | EGFR |
|  | 304 Y | | | 0.704 | unsp |  | 304 Y | 0.704 | unsp |
|  | 438 Y | | | 0.554 | unsp |  | 438 Y | 0.554 | unsp |
|  | 494 Y | | | 0.740 | unsp |  | 494 Y | 0.740 | unsp |
|  | 509 Y | | | 0.851 | unsp |  | 509 Y | 0.851 | unsp |
|  | 544 Y | | | 0.519 | EGFR |  | 544 Y | 0.519 | EGFR |
|  | 544 Y | | | 0.503 | INSR |  | 544 Y | 0.503 | INSR |
|  | 680 Y | | | 0.879 | unsp |  | 666 Y | 0.506 | INSR |
|  | 680 Y | | | 0.503 | INSR |  | 680 Y | 0.879 | unsp |
|  |  | | |  |  |  | 680 Y | 0.503 | INSR |
