# Supplementary material for: Computational investigation unveils pathogenic LIG3 non-synonymous mutations and therapeutic targets in acute myeloid leukemia
Source: PLoS One. 2025 Jun 10;20(6):e0320550. doi: 10.1371/journal.pone.0320550 (PMC12151348; doi:10.1371/journal.pone.0320550)
Supplement: S5 Table — (DOCX) [file pone.0320550.s005.docx]

**S5 Table:** Structural alterations, mutations in conserved domains, and amino acid characteristics of the wild-type and mutant-type amino acids through project hope.

| Amino acid change | Structure | Mutation located in the conserved domain | Conservation  (located near a highly conserved position) | Amino acid properties |
| --- | --- | --- | --- | --- |
| R528C | 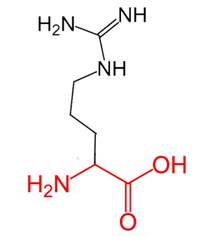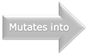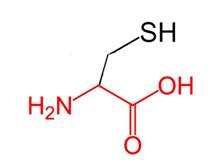 | Yes | Yes | - The wild-type residue charge was POSITIVE, the mutant residue charge is NEUTRAL. This can cause loss of interactions with other molecules. - The mutant residue is more hydrophobic than the wild-type residue. - The mutant residue is smaller than the wild-type residue. This will cause a possible loss of external interactions. |
| R671G | 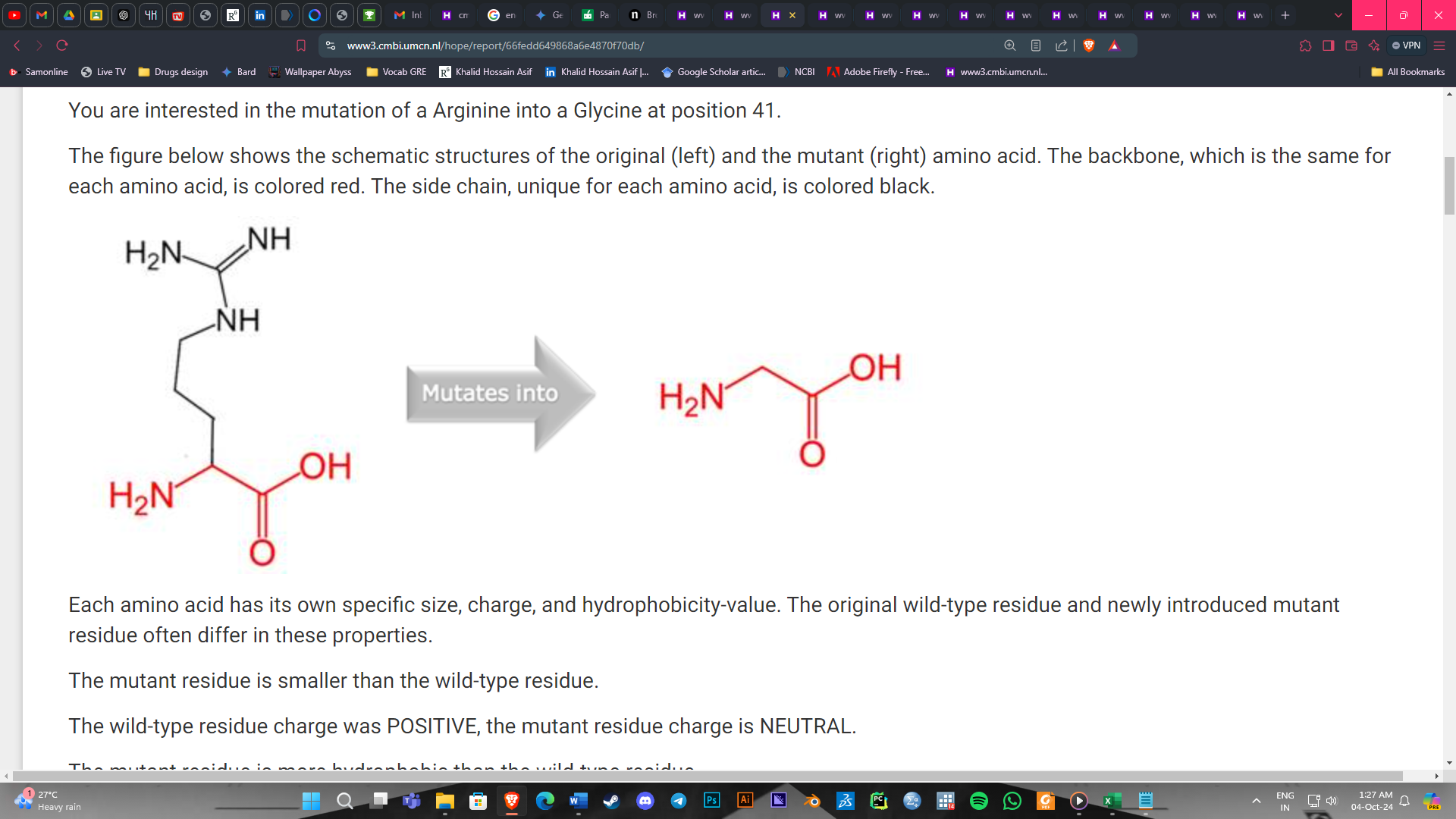 | Yes | No | - The wild-type residue charge was POSITIVE, the mutant residue charge is NEUTRAL. - The mutant residue is more hydrophobic than the wild-type residue. - The wild-type and mutant amino acids differ in size. - The mutant residue is smaller than the wild-type residue. |
| V781M | 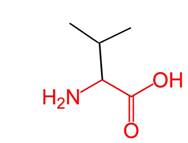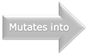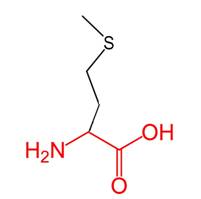 | Yes | Yes | - The wild-type and mutant amino acids differ in size. - The mutant residue is bigger than the wild-type residue, this might lead to bumps. - The wild-type residue is more hydrophobic than the mutant residue. - Hydrophobic interactions, either in the core of the protein or on the surface, will be lost. |
| R528H | 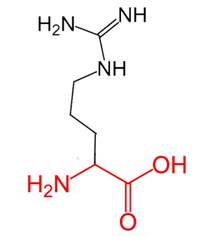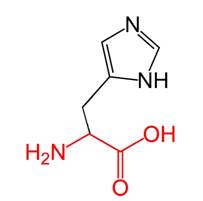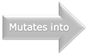 | Yes | No | - The wild-type and mutant amino acids differ in size. - The mutant residue is smaller than the wild-type residue, this might lead to loss of interactions. - The wild-type residue charge was POSITIVE, the mutant residue charge is NEUTRAL. - The charge of the wild-type residue will be lost, this can cause loss of interactions with other molecules or residues. |
| A432T | 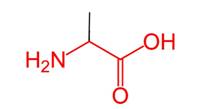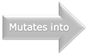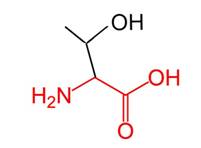 | Yes | Yes | - The wild-type and mutant amino acids differ in size. - The mutant residue is bigger than the wild-type residue, this might lead to bumps. - The wild-type residue is more hydrophobic than the mutant residue. - Hydrophobic interactions, either in the core of the protein or on the surface, will be lost. |
| L381R | 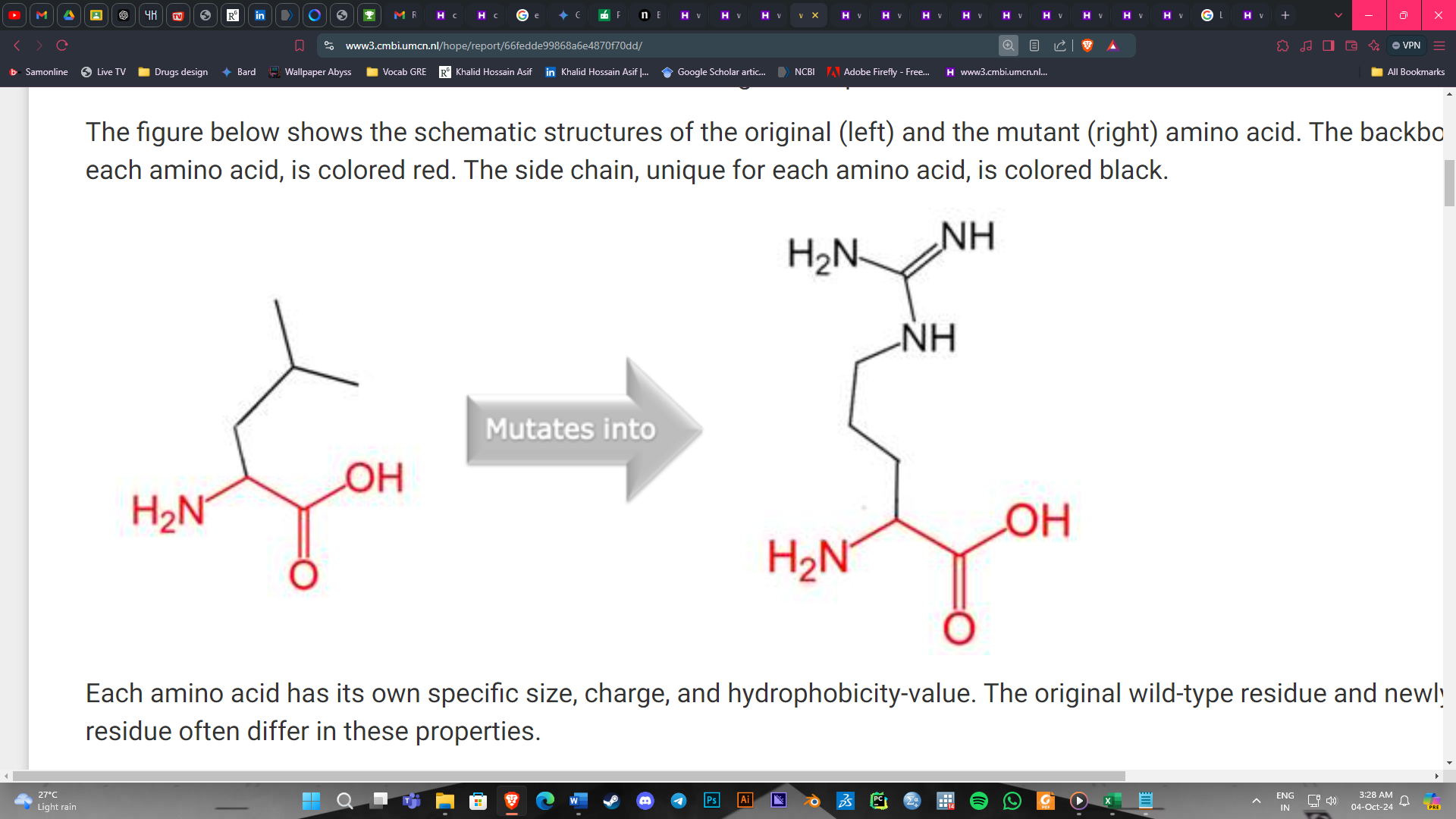 | Yes | No | - The wild-type and mutant amino acids differ in size. - The mutant residue is bigger than the wild-type residue. - The wild-type residue charge was NEUTRAL, the mutant residue charge is POSITIVE. - The wild-type residue is more hydrophobic than the mutant residue. |
| G799R | 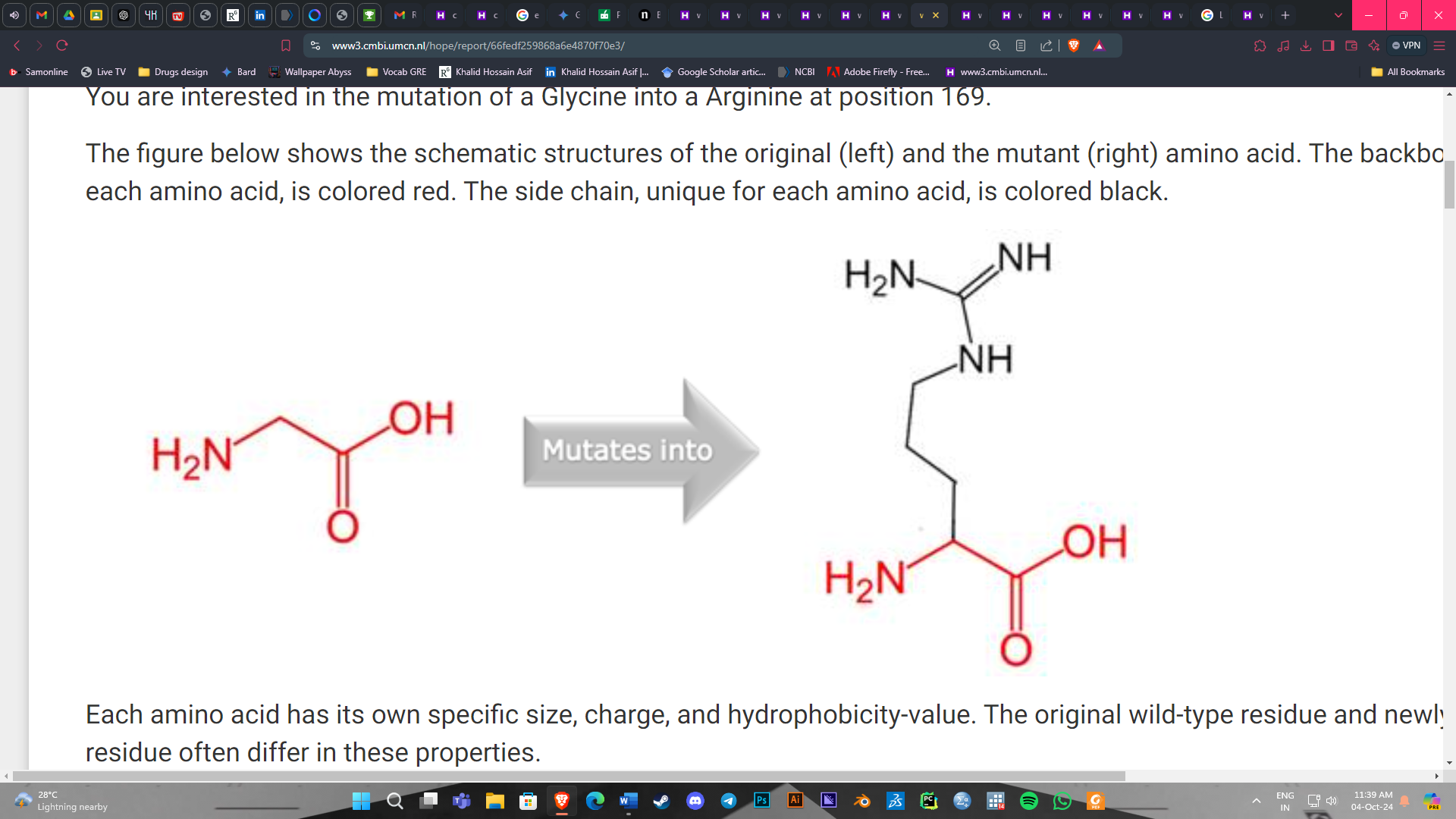 | Yes | No | - The wild-type and mutant amino acids differ in size. - The mutant residue is bigger than the wild-type residue. - The wild-type residue charge was NEUTRAL, the mutant residue charge is POSITIVE. - The wild-type residue is more hydrophobic than the mutant residue. |
| R806H | 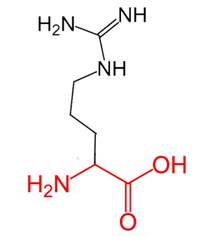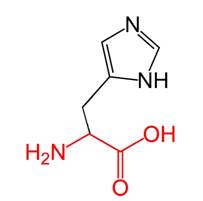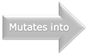 | Yes | No | - The wild-type and mutant amino acids differ in size. - The mutant residue is smaller than the wild-type residue, this might lead to loss of interactions. - The wild-type residue charge was POSITIVE, the mutant residue charge is NEUTRAL. - The charge of the wild-type residue will be lost, this can cause loss of interactions with other molecules or residues. |
| R614G | 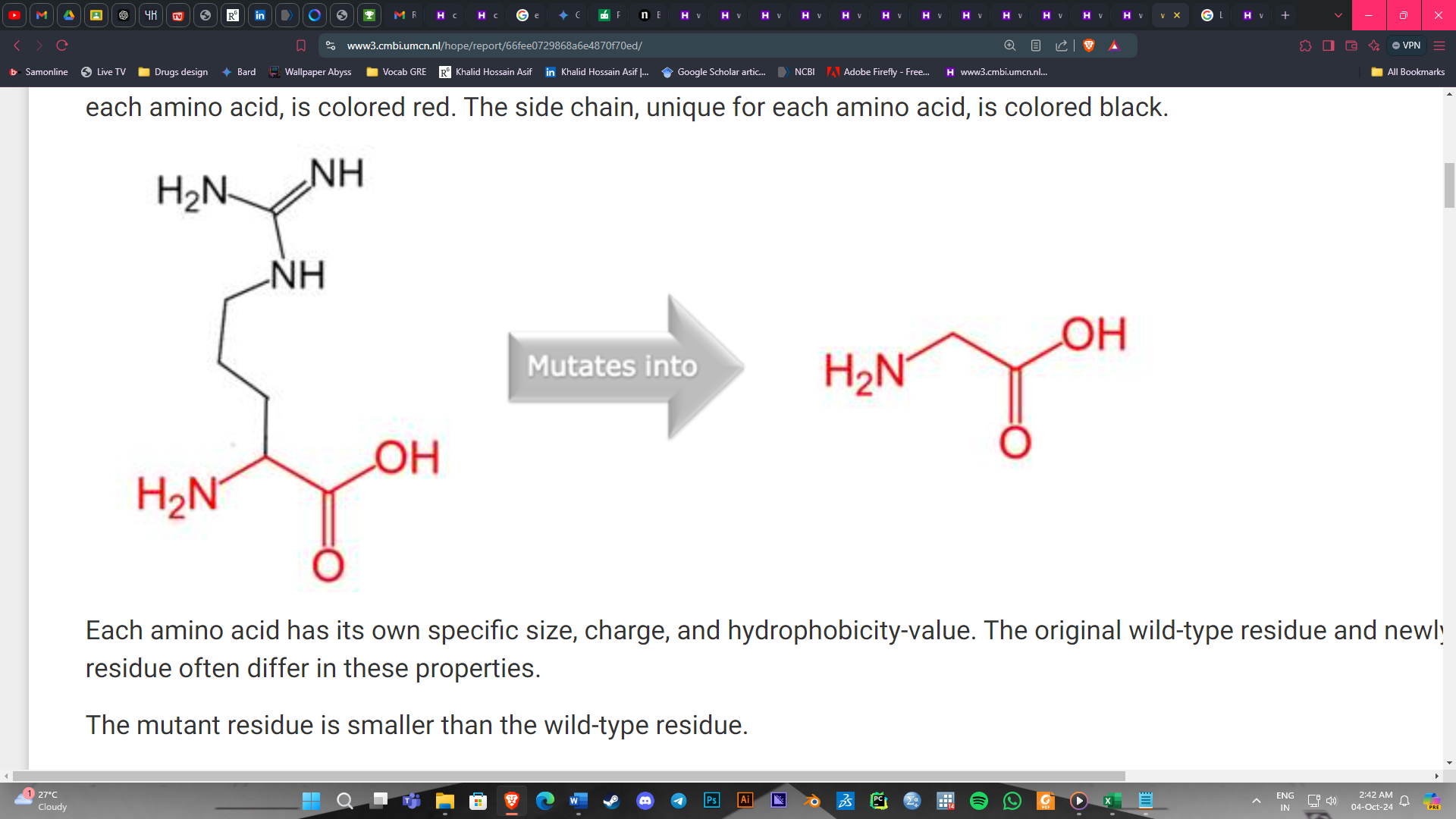 | Yes | Yes | - The wild-type and mutant amino acids differ in size. - The mutant residue is smaller than the wild-type residue. - The wild-type residue charge was POSITIVE, the mutant residue charge is NEUTRAL. - The mutant residue is more hydrophobic than the wild-type residue. |
