# Supplementary material for: Computational investigation unveils pathogenic LIG3 non-synonymous mutations and therapeutic targets in acute myeloid leukemia
Source: PLoS One. 2025 Jun 10;20(6):e0320550. doi: 10.1371/journal.pone.0320550 (PMC12151348; doi:10.1371/journal.pone.0320550)
Supplement: S6 Table — (DOCX) [file pone.0320550.s006.docx]

**S6 Table:**  Functions of proteins linked with *LIG3* gene in PPI.

| Serial no | Protein | Protein function |
| --- | --- | --- |
| 01 | DNA polymerase beta (POLB) | - POLB plays a central role in the Base Excision Repair (BER) pathway. - POLB works in conjunction with LIG3 to seal DNA nicks after filling gaps, ensuring the repair process is complete and accurate. |
| 02 | Aprataxin (APTX) | - APTX interacts with XRCC1-LIG3 complexes to enhance repair efficiency at single-strand break sites. - It removes adenylate groups from misactivated 5'-DNA ends, ensuring proper ligation by LIG3 or LIG1 during DNA repair. |
| 03 | Apurinic/Apyrimidinic Endodeoxyribonuclease 1 (APEX1) | - APEX1 is a key enzyme in BER, cleaving the phosphodiester backbone at abasic sites to create 3'-OH termini for downstream repair by LIG3 and other proteins. - APEX1 collaborates with LIG3 and XRCC1, forming a repair complex to efficiently resolve single-strand breaks during BER. |
| 04 | Aprataxin and PNK-like factor (APLF) | - APLF plays a critical role in processing DNA ends during non-homologous end joining (NHEJ), aiding in the preparation of DNA termini for ligation by LIG3 or LIG4. - Involved in single-strand and double-strand DNA break repair - Displays apurinic- apyrimidinic (AP) endonuclease and 3'-5' exonuclease activities in vitro |
| 05 | X-ray repair cross-complementing protein 6 (XRCC6) | - XRCC6 has a role in chromosome translocation by reducing the risk of chromosomal rearrangements or mutations. - Helps DNA helicase II complex to bind with DNA. Involved in DNA non-homologous end joining (NHEJ) required for double-strand break repair and V(D)J recombination. - XRCC6 helps recruit and organize the DNA-dependent protein kinase (DNA-PK) complex, including DNA-PKcs, and facilitates ligation by DNA ligases such as LIG3 and LIG4. |
| 06 | X-ray repair cross-complementing protein 1 (XRCC1) | - XRCC1 acts as a scaffold protein, coordinating the assembly of DNA repair complexes by directly interacting with LIG3 and other repair proteins to facilitate single-strand break (SSB) repair. - It localizes LIG3 to DNA damage sites through interactions with poly (ADP-ribose) polymerase (PARP) proteins, such as PARP1 and PARP2, promoting efficient DNA repair. |
| 07 | Tyrosyl-DNA phosphodiesterase 1 (TDP1) | - TDP1 works in conjunction with XRCC1 and LIG3 to facilitate the repair of single-strand breaks caused by stalled topoisomerase I activity. - It processes various 3'-blocking lesions, such as 3'-phosphoglycolates, created by oxidative damage, preparing DNA ends for ligation by LIG3. |
| 08 | Polynucleotide Kinase Phosphatase (PNKP) | - PNKP functions in both the base excision repair (BER) and non-homologous end joining (NHEJ) pathways, ensuring accurate repair of single- and double-strand breaks. - It interacts with XRCC1 to efficiently coordinate DNA repair processes, ensuring proper end processing before ligation by LIG3. |
| 09 | Poly [ADP-ribose] polymerase 1 (PARP1) | - PARP1 plays a key role in DNA repair processes. - Mediates glutamate and aspartate ADP-ribosylation of target proteins: the ADP-D- ribosyl group of NAD (+) is transferred to the acceptor carboxyl group of glutamate and aspartate residues and further ADP-ribosyl groups are transferred to the 2'-position of the terminal adenosine moiety, building up a polymer with an average chain length of 20-30 units. - Mediates the poly ADP-ribosylation of a number of proteins |
| 10 | Poly [ADP-ribose] polymerase 2 (PARP2) | - PARP2 modifies chromatin structure through poly-ADP ribosylation, promoting the accessibility of repair proteins like XRCC1 and LIG3 to damaged DNA sites. - It is involved in the BER pathway, enhancing LIG3's ligation activity for sealing DNA nicks during the repair of damaged bases. |
