# Supplementary material for: Computational investigation unveils pathogenic LIG3 non-synonymous mutations and therapeutic targets in acute myeloid leukemia
Source: PLoS One. 2025 Jun 10;20(6):e0320550. doi: 10.1371/journal.pone.0320550 (PMC12151348; doi:10.1371/journal.pone.0320550)
Supplement: S7 Table — (DOCX) [file pone.0320550.s007.docx]

| **Ligands** | Wild-type  *LIG3* | Mutant-type  R671G | Mutant-type  V781M | Mutant-type  R528C | Interacting residues | | | | | | | | |
| --- | --- | --- | --- | --- | --- | --- | --- | --- | --- | --- | --- | --- | --- |
|  |  |  |  |  | Hydrogen bond | | | | Hydrophobic bond | | | | |
|  |  |  |  |  | Wild-type  *LIG3* | Mutant-type  R528C | Mutant-type  R671G | Mutant-type V781M | Wild-type *LIG3* | Mutant-type R528C | Mutant-type  R671G | Mutant-type  V781M |  |
| 3-amino-N-(5-hydroxy-2-adamantyl)-6-[3-(1-methylpyrazol-4-yl)phenyl]pyrazine-2-carboxamide (AHP-MPC) : (CID: 70687578) | -10.2 | -8.4 | -7.8 | -9.4 | Tyr^509^ | Ala^486^, Lys^660^ | Arg^513^,  Lys^675^ | Leu^447^,  Arg^445^ | Glu^560^  Lys^675^  Leu^485^  Ala^486^  Lys^508^  Val^658^  Phe^595^ | Leu^485^,  Arg^513^,  Lys^508^,  Val^658^,  Glu^560^ | Val^658^,  Phe^595^,  Trp^673^,  Ala^486^,  Arg^528^,  Lys^508^,  Glu^655^ | Ile^315^,  Lys^531^ |  |
| 9,10-dimethyl-5H-benzo[c]furo[3,2-g]chromen-5-one (DM-BFC): (CID: 707801) | -9.2 | -9.3 | -9.3 | -9.9 | Asp^812^,  Lys^777^ | Arg^513^,  Glu^655^ | Asp^510^ | Asp^510^ | Leu^745^,  Trp^782^,  Leu^741^, | Lys^675^,  Lys^508^,  Ala^486^,  Val^658^ | Phe^574^,  Pro^573^,  Pro^571,^  Leu^654^ | Phe^574^,  Pro^571^,  Pro^573^,  Leu^654^ |  |

**S7 Table:** Analysis of the binding affinity of wild-type LIG3 assessed to its mutant variants, along with the associated interacting residues.
