# Supplementary material for: Computational investigation unveils pathogenic LIG3 non-synonymous mutations and therapeutic targets in acute myeloid leukemia
Source: PLoS One. 2025 Jun 10;20(6):e0320550. doi: 10.1371/journal.pone.0320550 (PMC12151348; doi:10.1371/journal.pone.0320550)
Supplement: S8 Table — (DOCX) [file pone.0320550.s008.docx]

**S8 Table:** Screening of 16 compounds that are associated with AML and several cancers.

| Ligands  (CID) | Wild-type  *LIG3* | Mutant-type  R528C | Mutant-type  R671G | Mutant-type  V781M |
| --- | --- | --- | --- | --- |
| 707801 | -10.2 | -9.4 | -8.4 | -7.8 |
| 70687578 | -9.2 | -9.9 | -9.3 | -9.3 |
| 59937 | -7.8 | -7.4 | -5.5 | -5.4 |
| 408383 | -7.0 | -6.2 | -6.1 | -5.9 |
| 676443 | -5.4 | -5.4 | -4.4 | -4.5 |
| 718154 | -7.8 | -7.6 | -6.4 | -6.4 |
| 722325 | -9.3 | -7.9 | -7.1 | -6.9 |
| 609964 | -7.7 | -7.0 | -6.0 | -6.0 |
| 116535 | -9.9 | -8.6 | -7.6 | -8.7 |
| 684700 | -7.6 | -7.4 | -6.9 | -6.1 |
| 627757 | -9.4 | -8.5 | -7.9 | -7.9 |
| 743508 | -8.4 | -7.8 | -6.3 | -6.4 |
| 695267 | -9.3 | -7.6 | -7.9 | -7.8 |
| 666597 | -7.5 | -7.1 | -6.4 | -6.2 |
| 743508 | -8.4 | -7.8 | -6.3 | -6.4 |
| 749518 | -6.9 | -8.6 | -6.6 | -7.0 |
