# Supplementary material for: Computational investigation unveils pathogenic LIG3 non-synonymous mutations and therapeutic targets in acute myeloid leukemia
Source: PLoS One. 2025 Jun 10;20(6):e0320550. doi: 10.1371/journal.pone.0320550 (PMC12151348; doi:10.1371/journal.pone.0320550)
Supplement: S9 Table — (DOCX) [file pone.0320550.s009.docx]

**S9 Table:** Summary of GEO profiles datasets used for ROC curve analysis comparing AML blast samples and mixed lineage leukemia (MLL) expression profiles.

| Sample | Title | Value | Rank |
| --- | --- | --- | --- |
| [GSM856203](https://www.ncbi.nlm.nih.gov/geo/query/acc.cgi?acc=GSM856203) | Acute Myeloid Leukemia, sample 1 | 585.3 | 75 |
| [GSM856204](https://www.ncbi.nlm.nih.gov/geo/query/acc.cgi?acc=GSM856204) | Acute Myeloid Leukemia, sample 2 | 426.1 | 69 |
| [GSM856205](https://www.ncbi.nlm.nih.gov/geo/query/acc.cgi?acc=GSM856205) | Acute Myeloid Leukemia, sample 3 | 413.8 | 68 |
| [GSM856206](https://www.ncbi.nlm.nih.gov/geo/query/acc.cgi?acc=GSM856206) | Acute Myeloid Leukemia, sample 4 | 431.1 | 70 |
| [GSM856207](https://www.ncbi.nlm.nih.gov/geo/query/acc.cgi?acc=GSM856207) | Acute Myeloid Leukemia, sample 5 | 310.1 | 65 |
| [GSM856208](https://www.ncbi.nlm.nih.gov/geo/query/acc.cgi?acc=GSM856208) | Acute Myeloid Leukemia, sample 6 | 46.6 | 30 |
| [GSM856209](https://www.ncbi.nlm.nih.gov/geo/query/acc.cgi?acc=GSM856209) | Acute Myeloid Leukemia, sample 7 | 413.9 | 69 |
| [GSM856210](https://www.ncbi.nlm.nih.gov/geo/query/acc.cgi?acc=GSM856210) | Acute Myeloid Leukemia, sample 8 | 218.6 | 56 |
| [GSM856211](https://www.ncbi.nlm.nih.gov/geo/query/acc.cgi?acc=GSM856211) | Acute Myeloid Leukemia, sample 9 | 314.4 | 64 |
| [GSM856212](https://www.ncbi.nlm.nih.gov/geo/query/acc.cgi?acc=GSM856212) | Acute Myeloid Leukemia, sample 10 | 173 | 49 |
| [GSM856213](https://www.ncbi.nlm.nih.gov/geo/query/acc.cgi?acc=GSM856213) | Acute Myeloid Leukemia, sample 11 | 271.9 | 66 |
| [GSM856214](https://www.ncbi.nlm.nih.gov/geo/query/acc.cgi?acc=GSM856214) | Acute Myeloid Leukemia, sample 12 | 164.8 | 55 |
| [GSM856215](https://www.ncbi.nlm.nih.gov/geo/query/acc.cgi?acc=GSM856215) | Acute Myeloid Leukemia, sample 13 | 235.5 | 59 |
| [GSM856216](https://www.ncbi.nlm.nih.gov/geo/query/acc.cgi?acc=GSM856216) | Acute Myeloid Leukemia, sample 14 | 445.9 | 70 |
| [GSM856217](https://www.ncbi.nlm.nih.gov/geo/query/acc.cgi?acc=GSM856217) | Acute Myeloid Leukemia, sample 15 | 274.3 | 59 |
| [GSM856218](https://www.ncbi.nlm.nih.gov/geo/query/acc.cgi?acc=GSM856218) | Acute Myeloid Leukemia, sample 16 | 431.4 | 70 |
| [GSM856219](https://www.ncbi.nlm.nih.gov/geo/query/acc.cgi?acc=GSM856219) | Acute Myeloid Leukemia, sample 17 | 128.8 | 49 |
| [GSM856220](https://www.ncbi.nlm.nih.gov/geo/query/acc.cgi?acc=GSM856220) | Acute Myeloid Leukemia, sample 18 | 81.3 | 35 |
| [GSM856221](https://www.ncbi.nlm.nih.gov/geo/query/acc.cgi?acc=GSM856221) | Acute Myeloid Leukemia, sample 19 | 218.2 | 60 |
| [GSM856222](https://www.ncbi.nlm.nih.gov/geo/query/acc.cgi?acc=GSM856222) | Acute Myeloid Leukemia, sample 20 | 332.3 | 67 |
| [GSM856223](https://www.ncbi.nlm.nih.gov/geo/query/acc.cgi?acc=GSM856223) | Acute Myeloid Leukemia, sample 21 | 195.2 | 58 |
| [GSM856224](https://www.ncbi.nlm.nih.gov/geo/query/acc.cgi?acc=GSM856224) | Acute Myeloid Leukemia, sample 22 | 385.7 | 68 |
| [GSM856225](https://www.ncbi.nlm.nih.gov/geo/query/acc.cgi?acc=GSM856225) | Acute Myeloid Leukemia, sample 23 | 375.2 | 69 |
| [GSM856226](https://www.ncbi.nlm.nih.gov/geo/query/acc.cgi?acc=GSM856226) | Acute Myeloid Leukemia, sample 24 | 321.6 | 66 |
| [GSM856227](https://www.ncbi.nlm.nih.gov/geo/query/acc.cgi?acc=GSM856227) | Acute Myeloid Leukemia, sample 25 | 343.7 | 67 |
| [GSM856228](https://www.ncbi.nlm.nih.gov/geo/query/acc.cgi?acc=GSM856228) | Acute Myeloid Leukemia, sample 26 | 348.6 | 68 |
| [GSM856229](https://www.ncbi.nlm.nih.gov/geo/query/acc.cgi?acc=GSM856229) | Acute Myeloid Leukemia, sample 27 | 238.7 | 61 |
| [GSM856230](https://www.ncbi.nlm.nih.gov/geo/query/acc.cgi?acc=GSM856230) | Acute Myeloid Leukemia, sample 28 | 203.9 | 58 |
| [GSM856231](https://www.ncbi.nlm.nih.gov/geo/query/acc.cgi?acc=GSM856231) | Acute Myeloid Leukemia, sample 29 | 66.8 | 36 |
| [GSM856232](https://www.ncbi.nlm.nih.gov/geo/query/acc.cgi?acc=GSM856232) | Acute Myeloid Leukemia, sample 30 | 206 | 57 |
| [GSM856233](https://www.ncbi.nlm.nih.gov/geo/query/acc.cgi?acc=GSM856233) | Acute Myeloid Leukemia, sample 31 | 79 | 36 |
| [GSM856234](https://www.ncbi.nlm.nih.gov/geo/query/acc.cgi?acc=GSM856234) | Acute Myeloid Leukemia, sample 32 | 164.5 | 54 |
| [GSM856235](https://www.ncbi.nlm.nih.gov/geo/query/acc.cgi?acc=GSM856235) | Acute Myeloid Leukemia, sample 33 | 303.7 | 64 |
| [GSM856236](https://www.ncbi.nlm.nih.gov/geo/query/acc.cgi?acc=GSM856236) | Acute Myeloid Leukemia, sample 34 | 251.6 | 64 |
| [GSM856237](https://www.ncbi.nlm.nih.gov/geo/query/acc.cgi?acc=GSM856237) | Acute Myeloid Leukemia, sample 35 | 168.3 | 50 |
| [GSM856238](https://www.ncbi.nlm.nih.gov/geo/query/acc.cgi?acc=GSM856238) | Acute Myeloid Leukemia, sample 36 | 272.7 | 63 |
| [GSM856239](https://www.ncbi.nlm.nih.gov/geo/query/acc.cgi?acc=GSM856239) | Acute Myeloid Leukemia, sample 37 | 59.8 | 27 |
| [GSM856240](https://www.ncbi.nlm.nih.gov/geo/query/acc.cgi?acc=GSM856240) | Acute Myeloid Leukemia, sample 38 | 434.5 | 72 |
| [GSM856241](https://www.ncbi.nlm.nih.gov/geo/query/acc.cgi?acc=GSM856241) | Acute Myeloid Leukemia, sample 39 | 121.2 | 46 |
| [GSM856242](https://www.ncbi.nlm.nih.gov/geo/query/acc.cgi?acc=GSM856242) | Acute Myeloid Leukemia, sample 40 | 190 | 58 |
| [GSM856243](https://www.ncbi.nlm.nih.gov/geo/query/acc.cgi?acc=GSM856243) | Acute Myeloid Leukemia, sample 41 | 210.4 | 54 |
| [GSM856244](https://www.ncbi.nlm.nih.gov/geo/query/acc.cgi?acc=GSM856244) | Acute Myeloid Leukemia, sample 42 | 173.4 | 55 |
| [GSM856245](https://www.ncbi.nlm.nih.gov/geo/query/acc.cgi?acc=GSM856245) | Acute Myeloid Leukemia, sample 43 | 234.5 | 59 |
| [GSM856246](https://www.ncbi.nlm.nih.gov/geo/query/acc.cgi?acc=GSM856246) | Acute Myeloid Leukemia, sample 44 | 294.2 | 63 |
| [GSM856247](https://www.ncbi.nlm.nih.gov/geo/query/acc.cgi?acc=GSM856247) | Acute Myeloid Leukemia, sample 45 | 40.7 | 21 |
| [GSM856248](https://www.ncbi.nlm.nih.gov/geo/query/acc.cgi?acc=GSM856248) | Acute Myeloid Leukemia, sample 46 | 244 | 62 |
| [GSM856249](https://www.ncbi.nlm.nih.gov/geo/query/acc.cgi?acc=GSM856249) | Acute Myeloid Leukemia, sample 47 | 413.2 | 68 |
| [GSM856250](https://www.ncbi.nlm.nih.gov/geo/query/acc.cgi?acc=GSM856250) | Acute Myeloid Leukemia, sample 48 | 244.6 | 62 |
| [GSM856251](https://www.ncbi.nlm.nih.gov/geo/query/acc.cgi?acc=GSM856251) | Acute Myeloid Leukemia, sample 49 | 115.7 | 43 |
| [GSM856252](https://www.ncbi.nlm.nih.gov/geo/query/acc.cgi?acc=GSM856252) | Acute Myeloid Leukemia, sample 50 | 136.6 | 52 |
| [GSM856253](https://www.ncbi.nlm.nih.gov/geo/query/acc.cgi?acc=GSM856253) | Acute Myeloid Leukemia, sample 51 | 50.5 | 27 |
| [GSM856254](https://www.ncbi.nlm.nih.gov/geo/query/acc.cgi?acc=GSM856254) | Acute Myeloid Leukemia, sample 52 | 70.5 | 32 |
| [GSM856255](https://www.ncbi.nlm.nih.gov/geo/query/acc.cgi?acc=GSM856255) | Acute Myeloid Leukemia, sample 53 | 489.4 | 72 |
| [GSM856256](https://www.ncbi.nlm.nih.gov/geo/query/acc.cgi?acc=GSM856256) | Acute Myeloid Leukemia, sample 54 | 60.4 | 32 |
| [GSM856257](https://www.ncbi.nlm.nih.gov/geo/query/acc.cgi?acc=GSM856257) | Acute Myeloid Leukemia, sample 55 | 289.7 | 67 |
| [GSM856258](https://www.ncbi.nlm.nih.gov/geo/query/acc.cgi?acc=GSM856258) | Acute Myeloid Leukemia, sample 56 | 308.8 | 64 |
| [GSM856259](https://www.ncbi.nlm.nih.gov/geo/query/acc.cgi?acc=GSM856259) | Acute Myeloid Leukemia, sample 57 | 370.9 | 71 |
| [GSM856260](https://www.ncbi.nlm.nih.gov/geo/query/acc.cgi?acc=GSM856260) | Acute Myeloid Leukemia, sample 58 | 210.3 | 60 |
| [GSM856261](https://www.ncbi.nlm.nih.gov/geo/query/acc.cgi?acc=GSM856261) | Acute Myeloid Leukemia, sample 59 | 287.6 | 63 |
| [GSM856262](https://www.ncbi.nlm.nih.gov/geo/query/acc.cgi?acc=GSM856262) | Acute Myeloid Leukemia, sample 60 | 37.4 | 24 |
| [GSM856263](https://www.ncbi.nlm.nih.gov/geo/query/acc.cgi?acc=GSM856263) | Acute Myeloid Leukemia, sample 61 | 180.7 | 58 |
| [GSM856264](https://www.ncbi.nlm.nih.gov/geo/query/acc.cgi?acc=GSM856264) | Acute Myeloid Leukemia, sample 62 | 86.7 | 36 |
| [GSM856265](https://www.ncbi.nlm.nih.gov/geo/query/acc.cgi?acc=GSM856265) | Acute Myeloid Leukemia, sample 63 | 233.2 | 60 |
| [GSM856266](https://www.ncbi.nlm.nih.gov/geo/query/acc.cgi?acc=GSM856266) | Acute Myeloid Leukemia, sample 64 | 55.4 | 26 |
| [GSM856267](https://www.ncbi.nlm.nih.gov/geo/query/acc.cgi?acc=GSM856267) | Acute Myeloid Leukemia, sample 65 | 99.3 | 42 |
| [GSM856268](https://www.ncbi.nlm.nih.gov/geo/query/acc.cgi?acc=GSM856268) | Acute Myeloid Leukemia, sample 66 | 286.6 | 67 |
| [GSM856269](https://www.ncbi.nlm.nih.gov/geo/query/acc.cgi?acc=GSM856269) | Acute Myeloid Leukemia, sample 67 | 228.9 | 59 |
| [GSM856270](https://www.ncbi.nlm.nih.gov/geo/query/acc.cgi?acc=GSM856270) | Acute Myeloid Leukemia, sample 68 | 455.8 | 73 |
| [GSM856271](https://www.ncbi.nlm.nih.gov/geo/query/acc.cgi?acc=GSM856271) | Acute Myeloid Leukemia, sample 69 | 118.7 | 48 |
| [GSM856272](https://www.ncbi.nlm.nih.gov/geo/query/acc.cgi?acc=GSM856272) | Acute Myeloid Leukemia, sample 70 | 220.8 | 63 |
| [GSM856273](https://www.ncbi.nlm.nih.gov/geo/query/acc.cgi?acc=GSM856273) | Acute Myeloid Leukemia, sample 71 | 84.7 | 38 |
| [GSM856274](https://www.ncbi.nlm.nih.gov/geo/query/acc.cgi?acc=GSM856274) | Acute Myeloid Leukemia, sample 72 | 296.4 | 64 |
| [GSM856275](https://www.ncbi.nlm.nih.gov/geo/query/acc.cgi?acc=GSM856275) | Acute Myeloid Leukemia, sample 73 | 73.4 | 36 |
| [GSM856276](https://www.ncbi.nlm.nih.gov/geo/query/acc.cgi?acc=GSM856276) | Acute Myeloid Leukemia, sample 74 | 459.6 | 71 |
| [GSM856277](https://www.ncbi.nlm.nih.gov/geo/query/acc.cgi?acc=GSM856277) | Acute Myeloid Leukemia, sample 75 | 53.5 | 26 |
| [GSM856278](https://www.ncbi.nlm.nih.gov/geo/query/acc.cgi?acc=GSM856278) | Acute Myeloid Leukemia, sample 76 | 79.9 | 33 |
| [GSM856279](https://www.ncbi.nlm.nih.gov/geo/query/acc.cgi?acc=GSM856279) | Acute Myeloid Leukemia, sample 77 | 247.7 | 61 |
| [GSM856280](https://www.ncbi.nlm.nih.gov/geo/query/acc.cgi?acc=GSM856280) | Acute Myeloid Leukemia, sample 78 | 93.1 | 38 |
| [GSM304968](https://www.ncbi.nlm.nih.gov/geo/query/acc.cgi?acc=GSM304968) | tMLL01 | 258.003 | 65 |
| [GSM307951](https://www.ncbi.nlm.nih.gov/geo/query/acc.cgi?acc=GSM307951) | tMLL02 | 393.221 | 71 |
| [GSM307952](https://www.ncbi.nlm.nih.gov/geo/query/acc.cgi?acc=GSM307952) | tMLL03 | 152.371 | 54 |
| [GSM307953](https://www.ncbi.nlm.nih.gov/geo/query/acc.cgi?acc=GSM307953) | tMLL04 | 367.841 | 68 |
| [GSM307957](https://www.ncbi.nlm.nih.gov/geo/query/acc.cgi?acc=GSM307957) | tMLL05 | 434.428 | 73 |
| [GSM307958](https://www.ncbi.nlm.nih.gov/geo/query/acc.cgi?acc=GSM307958) | tMLL06 | 426.705 | 71 |
| [GSM307959](https://www.ncbi.nlm.nih.gov/geo/query/acc.cgi?acc=GSM307959) | tMLL07 | 413.653 | 70 |
| [GSM307960](https://www.ncbi.nlm.nih.gov/geo/query/acc.cgi?acc=GSM307960) | tMLL08 | 211.057 | 61 |
| [GSM307961](https://www.ncbi.nlm.nih.gov/geo/query/acc.cgi?acc=GSM307961) | tMLL09 | 184.137 | 60 |
| [GSM307966](https://www.ncbi.nlm.nih.gov/geo/query/acc.cgi?acc=GSM307966) | tMLL10 | 390.426 | 71 |
| [GSM366160](https://www.ncbi.nlm.nih.gov/geo/query/acc.cgi?acc=GSM366160) | MLL_PTD01 | 416.956 | 70 |
| [GSM366161](https://www.ncbi.nlm.nih.gov/geo/query/acc.cgi?acc=GSM366161) | MLL_PTD02 | 147.071 | 45 |
| [GSM366162](https://www.ncbi.nlm.nih.gov/geo/query/acc.cgi?acc=GSM366162) | MLL_PTD03 | 344.299 | 68 |
| [GSM366163](https://www.ncbi.nlm.nih.gov/geo/query/acc.cgi?acc=GSM366163) | MLL_PTD04 | 340.255 | 68 |
| [GSM366164](https://www.ncbi.nlm.nih.gov/geo/query/acc.cgi?acc=GSM366164) | MLL_PTD05 | 193.196 | 58 |
| [GSM366165](https://www.ncbi.nlm.nih.gov/geo/query/acc.cgi?acc=GSM366165) | MLL_PTD06 | 305.312 | 66 |
| [GSM366166](https://www.ncbi.nlm.nih.gov/geo/query/acc.cgi?acc=GSM366166) | MLL_PTD07 | 302.112 | 68 |
| [GSM366167](https://www.ncbi.nlm.nih.gov/geo/query/acc.cgi?acc=GSM366167) | MLL_PTD08 | 430.567 | 72 |
| [GSM366168](https://www.ncbi.nlm.nih.gov/geo/query/acc.cgi?acc=GSM366168) | MLL_PTD09 | 238.835 | 64 |
| [GSM366169](https://www.ncbi.nlm.nih.gov/geo/query/acc.cgi?acc=GSM366169) | MLL_PTD10 | 177.689 | 59 |
| [GSM366170](https://www.ncbi.nlm.nih.gov/geo/query/acc.cgi?acc=GSM366170) | MLL_PTD11 | 307.845 | 68 |
| [GSM366171](https://www.ncbi.nlm.nih.gov/geo/query/acc.cgi?acc=GSM366171) | MLL_PTD12 | 320.804 | 66 |
| [GSM366172](https://www.ncbi.nlm.nih.gov/geo/query/acc.cgi?acc=GSM366172) | MLL_PTD13 | 345.087 | 69 |
| [GSM366173](https://www.ncbi.nlm.nih.gov/geo/query/acc.cgi?acc=GSM366173) | MLL_PTD14 | 362.963 | 72 |
| [GSM366174](https://www.ncbi.nlm.nih.gov/geo/query/acc.cgi?acc=GSM366174) | MLL_PTD15 | 304.34 | 69 |

**S1 Fig:**  A clustered pyramid visually depicts the quantity and arrangement of SNPs within the human *LIG3* gene, sourced from the dbSNP database (nsSNPs: 902; synonymous SNPs: 398; intronic SNPs: 9685; nsSNPs +Somatic: 132; others: 1074).

**
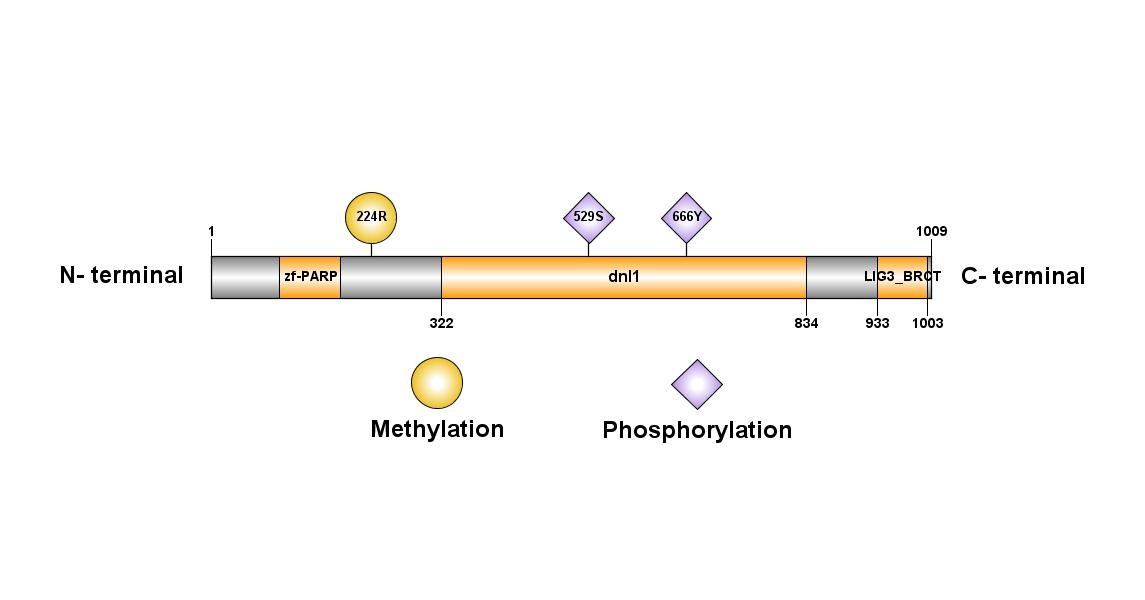
**

**S2 Fig:** Possible targeted phosphorylation and methylation sites as anticipated by GPS-MSP 1.0 and NetPhos 3.1 (using IBS software).

**
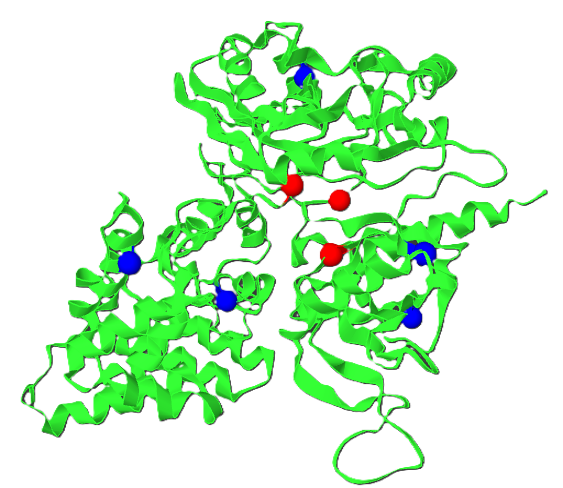
**

**S3 Fig:** The mutation 3D server identified certain nsSNPs as potential cancer-causing mutations (red mark). Red indicates clustered mutations, whereas blue signifies covered mutations. A represents cluster-1, where five nsSNPs (L381R, A432T, R614G, G799R, and R806H) are present, and B represents cluster-2 where four nsSNPs are (R528C, R528H, V781M, and R671G) present related with cancer (red mark).

**
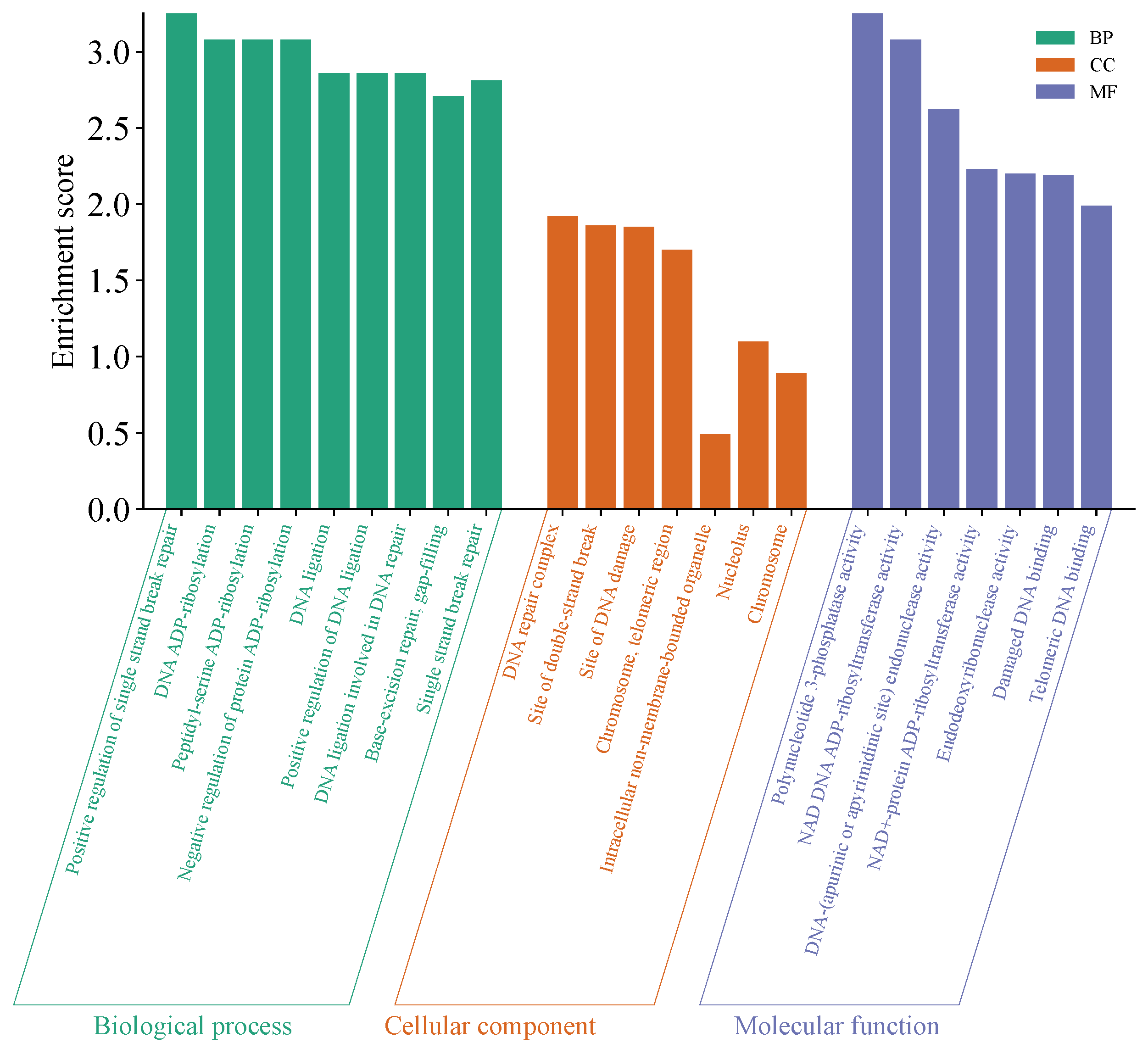
**

**S4 Fig:** Assessment of the LIG3 gene with a deep focus on Gene Ontology (GO) pathways, specifically Biological Process (BP), Cellular Component (CC), and Molecular Function (MF).

**
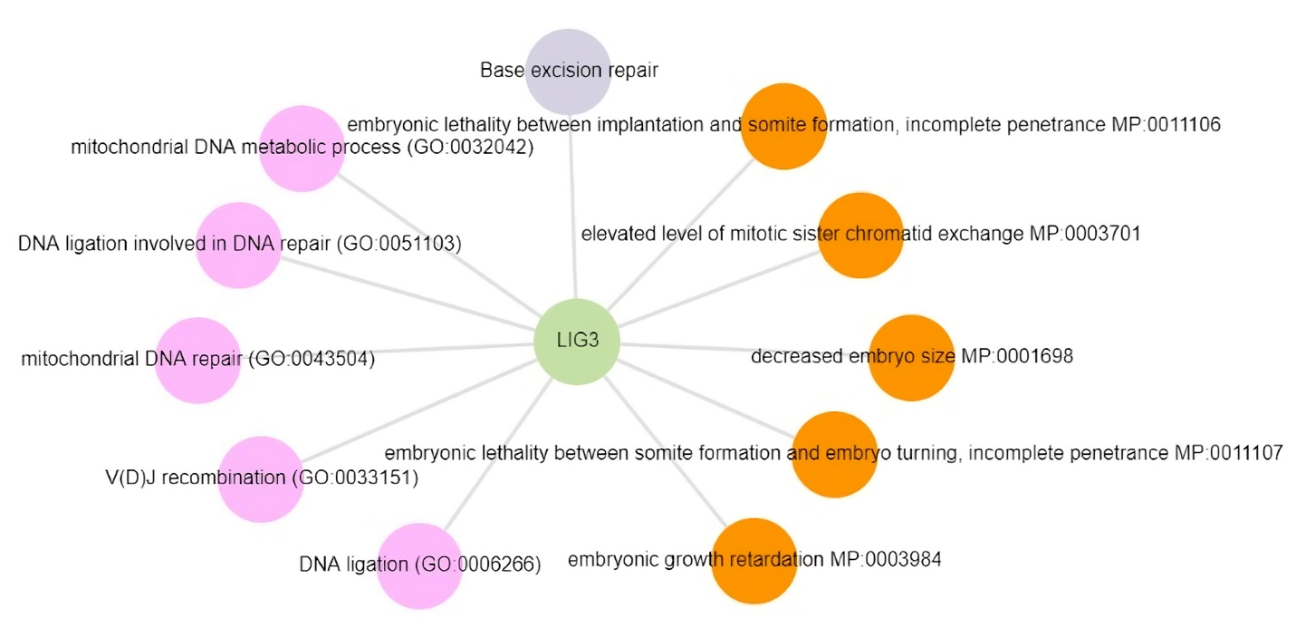
**

**S5 Fig:** Significant KEGG pathways of *LIG3* were represented in network view. The findings for the pathway term results were sorted based on the combined score (P-value).


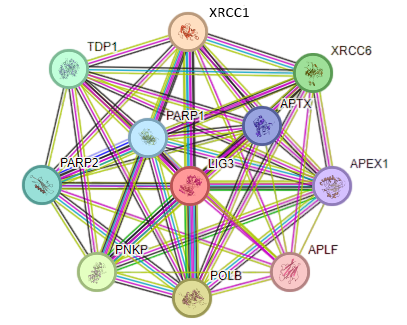


**S6 Fig:** STRING database analyzes PPI networking of LIG3 protein. Its straight line represents the connection between the proteins, while its circular form represents the proteins that are adjacent.
