# Supplementary material for: Computational investigation unveils pathogenic LIG3 non-synonymous mutations and therapeutic targets in acute myeloid leukemia
Source: PLoS One. 2025 Jun 10;20(6):e0320550. doi: 10.1371/journal.pone.0320550 (PMC12151348; doi:10.1371/journal.pone.0320550)
Supplement: S1 Fig — (DOCX) [file pone.0320550.s010.docx]

**S1 Fig:**  A clustered pyramid visually depicts the quantity and arrangement of SNPs within the human *LIG3* gene, sourced from the dbSNP database (nsSNPs: 902; synonymous SNPs: 398; intronic SNPs: 9685; nsSNPs +Somatic: 132; others: 1074).
