# Supplementary material for: Computational investigation unveils pathogenic LIG3 non-synonymous mutations and therapeutic targets in acute myeloid leukemia
Source: PLoS One. 2025 Jun 10;20(6):e0320550. doi: 10.1371/journal.pone.0320550 (PMC12151348; doi:10.1371/journal.pone.0320550)
Supplement: S2 Fig — (DOCX) [file pone.0320550.s011.docx]

**
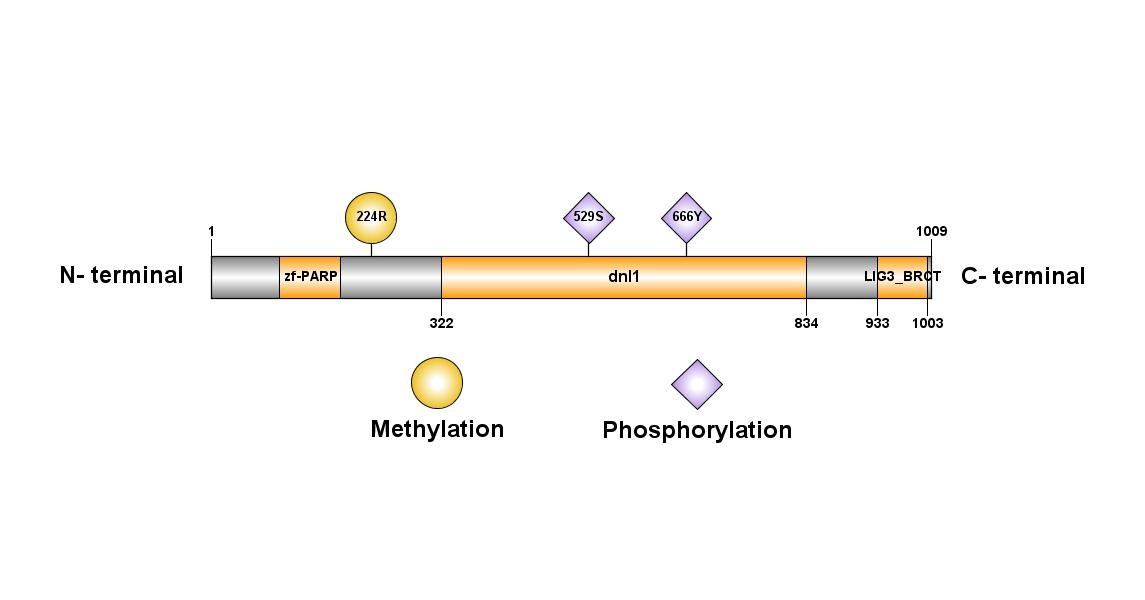
**

**S2 Fig:** Possible targeted phosphorylation and methylation sites as anticipated by GPS-MSP 1.0 and NetPhos 3.1 (using IBS software).
