# Supplementary material for: Computational investigation unveils pathogenic LIG3 non-synonymous mutations and therapeutic targets in acute myeloid leukemia
Source: PLoS One. 2025 Jun 10;20(6):e0320550. doi: 10.1371/journal.pone.0320550 (PMC12151348; doi:10.1371/journal.pone.0320550)
Supplement: S3 Fig — Red indicates clustered mutations, whereas blue signifies covered mutations. A represents cluster-1, where five nsSNPs (L381R, A432T, R614G, G799R, and R806H) are present, and B represents cluster-2 where four nsSNPs are (R528C, R528H, V781M, and R671G) present related with cancer (red mark). (DOCX) [file pone.0320550.s012.docx]

**
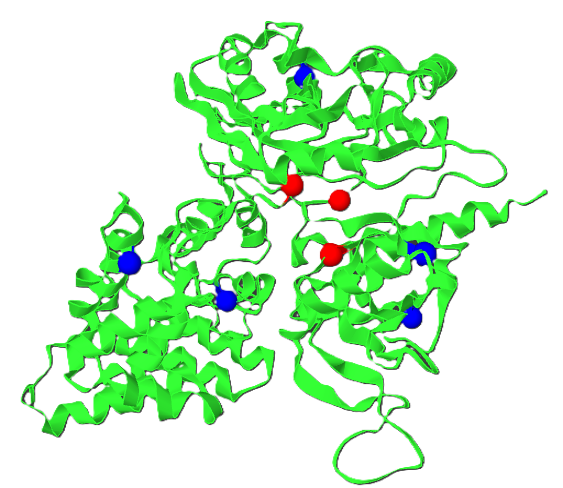
**

**S3 Fig:** The mutation 3D server identified certain nsSNPs as potential cancer-causing mutations (red mark). Red indicates clustered mutations, whereas blue signifies covered mutations. A represents cluster-1, where five nsSNPs (L381R, A432T, R614G, G799R, and R806H) are present, and B represents cluster-2 where four nsSNPs are (R528C, R528H, V781M, and R671G) present related with cancer (red mark).
