# Supplementary material for: Computational investigation unveils pathogenic LIG3 non-synonymous mutations and therapeutic targets in acute myeloid leukemia
Source: PLoS One. 2025 Jun 10;20(6):e0320550. doi: 10.1371/journal.pone.0320550 (PMC12151348; doi:10.1371/journal.pone.0320550)
Supplement: S4 Fig — (DOCX) [file pone.0320550.s013.docx]

**
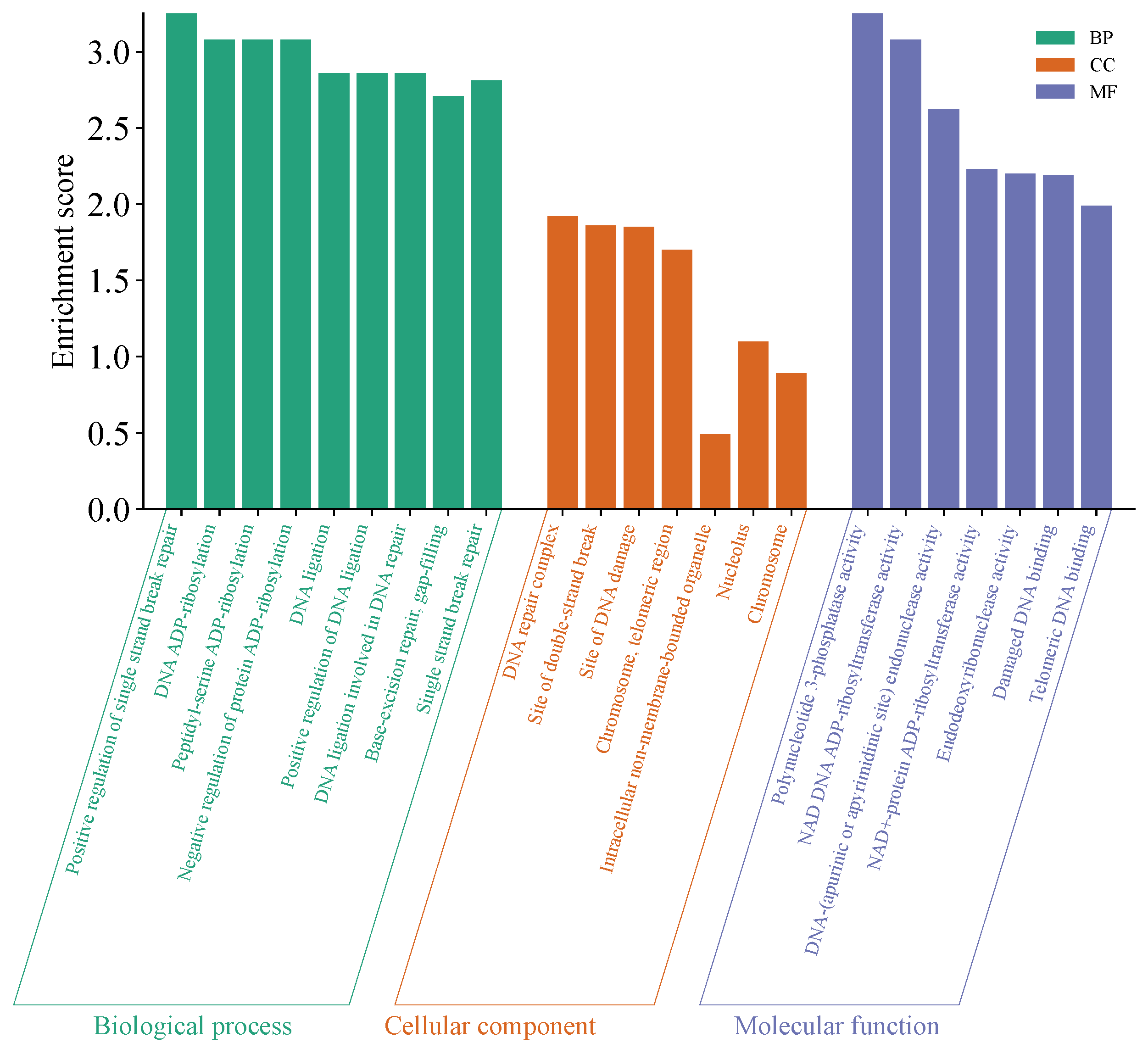
**

**S4 Fig:** Assessment of the LIG3 gene with a deep focus on Gene Ontology (GO) pathways, specifically Biological Process (BP), Cellular Component (CC), and Molecular Function (MF).
