# Supplementary material for: Computational investigation unveils pathogenic LIG3 non-synonymous mutations and therapeutic targets in acute myeloid leukemia
Source: PLoS One. 2025 Jun 10;20(6):e0320550. doi: 10.1371/journal.pone.0320550 (PMC12151348; doi:10.1371/journal.pone.0320550)
Supplement: S5 Fig — The findings for the pathway term results were sorted based on the combined score (P-value). (DOCX) [file pone.0320550.s014.docx]

**
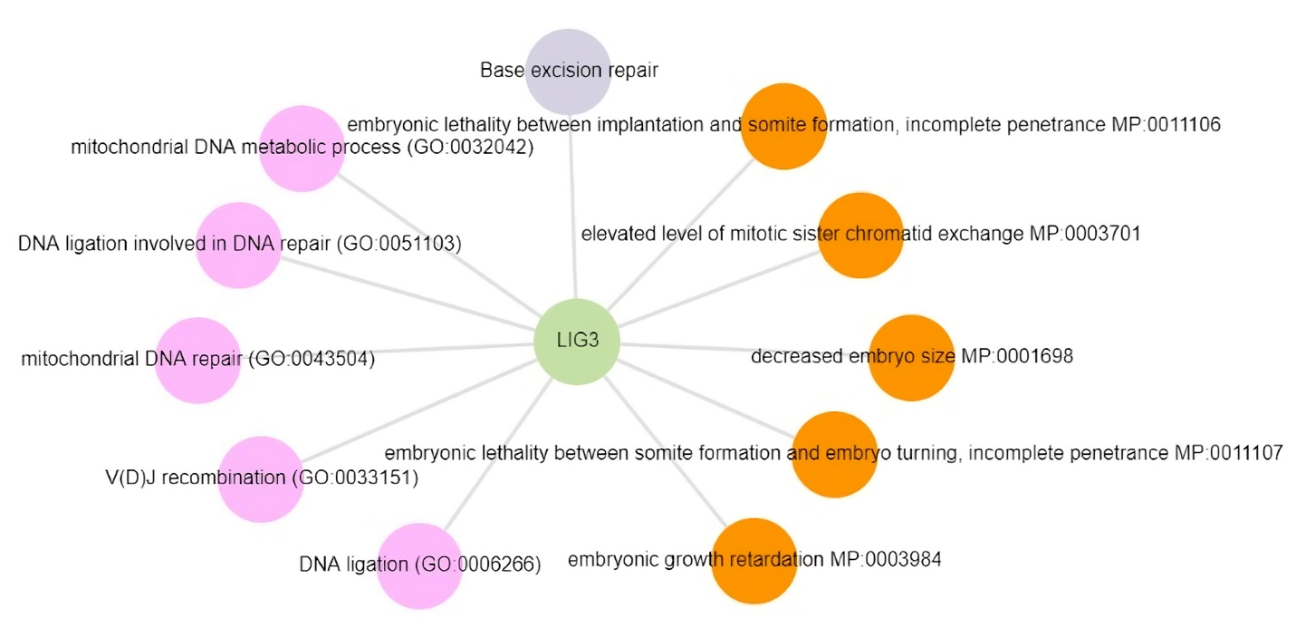
**

**S5 Fig:** Significant KEGG pathways of *LIG3* were represented in network view. The findings for the pathway term results were sorted based on the combined score (P-value).
