# Supplementary material for: Computational investigation unveils pathogenic LIG3 non-synonymous mutations and therapeutic targets in acute myeloid leukemia
Source: PLoS One. 2025 Jun 10;20(6):e0320550. doi: 10.1371/journal.pone.0320550 (PMC12151348; doi:10.1371/journal.pone.0320550)
Supplement: S6 Fig — Its straight line represents the connection between the proteins, while its circular form represents the proteins that are adjacent. (DOCX) [file pone.0320550.s015.docx]

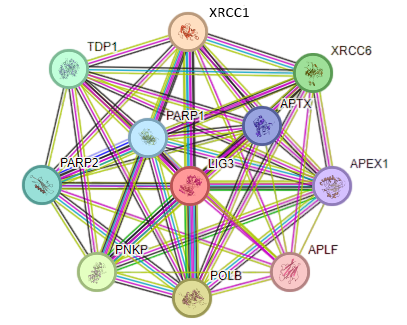


**S6 Fig:** STRING database analyzes PPI networking of LIG3 protein. Its straight line represents the connection between the proteins, while its circular form represents the proteins that are adjacent.
